# Legislation governing genetically modified and genome‐edited crops in Europe: the need for change

**DOI:** 10.1002/jsfa.9227

**Published:** 2018-08-19

**Authors:** Nigel G Halford

**Affiliations:** ^1^ Plant Sciences Department Rothamsted Research Harpenden UK

**Keywords:** genetic modification, genome editing, plant biotechnology, crop composition, regulation

## Abstract

The European Commission's assessment and approval process for genetically modified (GM) crops has resulted in only two GM crop varieties being licensed for cultivation in the European Union, one of which has been withdrawn. Unable to define GM crops satisfactorily, the European Commission has fallen back on a definition based on process. The shortcomings of this approach are all too clear as the Commission grapples with the advent of genome editing. This has led to a long and damaging delay in the Commission issuing an opinion on how genome‐edited crops should be regulated. At the same time, national bans imposed by member states on GM crops without any evidence of safety concerns have been legalized. The Commission also faces the prospect of assessing an increasing number of GM and genome‐edited crops with deliberately altered composition. In this article, the operation of regulations covering GM crops in the European Union and the effect they have had on the development of plant biotechnology are reviewed, while the issues raised by new technologies are discussed. It is argued that there is an urgent need for the European Union to shift its position on plant biotechnology if agriculture is to meet the challenges of coming decades. © 2018 The Author. *Journal of the Science of Food and Agriculture* published by John Wiley & Sons Ltd on behalf of Society of Chemical Industry.

## INTRODUCTION

Directive 2001/18/EC of the European Parliament and Council on the deliberate release into the environment of genetically modified organisms (GMOs), together with GM Food and Feed Regulation (EC) No. 1829/2003, which was adopted in 2004, brought the regulation of genetically modified (GM) crop use and release under the control of the European Commission. The European Union (EU) recognizes two different types of field release of GM crops: one for research purposes only (a Part B release) and one for commercial release (a Part C release). Consent for a Part C release covers cultivation, food and feed use, but it is also possible to apply for permission for food and feed use alone (i.e., for import and consumption in the EU but not for cultivation). Permission for a Part B release can be granted by an individual member state. However, applications for commercial use of GM crops or crop products anywhere in the EU have to be approved by the European Commission. Any food that incorporates a GM crop product also has to comply with stringent labelling laws.

This review comprises the author's opinion on how the regulations on GM crops in the EU have operated, what effect they have had on the development of plant biotechnology not only in Europe but also the rest of the world, recent changes that have been introduced, and how regulations will be applied to genome‐edited crops and GM crops with deliberately altered composition.

## THE EU ASSESSMENT AND APPROVAL PROCESS

An organization applying for a Part C consent to cultivate a GM crop in the EU must provide detailed information on the host plant species, as well as the nature of the genetic modification it carries and the methods used to bring the modification about. It must also undertake an assessment of the potential risks posed to health and/or the environment. This process begins with a comparison between the GM plant or food and its closest traditional counterpart in order to establish substantial equivalence and identify any intended and unintended differences. These differences then become the focus of the safety assessment and, if necessary, further investigation. Factors taken into account in the safety assessment include the identity and source of novel genes, the stability and potential for transfer of the novel gene or genes, the nature of the protein encoded by the novel gene or genes, potential changes in function of novel genes and proteins in the host background, the composition of the plant and/or food derived from it compared with its traditional counterpart, the effects of processing and cooking, potential toxicity or allergenicity of novel proteins, possible secondary effects resulting from expression of the novel gene or genes, and the potential intake and dietary impact of the introduction of the GM food. Applications for cultivation would also include an environmental impact assessment, and this would be carried out by a designated member state authority. Guidance can be found in the *Codex Alimentarius*, the annex to Commission Implementing Regulation (EU) No. 503/2013 and Directive (EU) 2018/350 (a recent amendment to Directive 2001/18/EC).

Each application is assessed by the European Food Safety Authority (EFSA) Genetically Modified Organisms (GMO) panel. If the GMO panel's opinion is favourable, the application is voted on by the Commission's Standing Committee on Plants, Animals, Food and Feed (PAFF), with representatives from all 28 member states. A qualified majority voting system is applied, in which approval requires 55% of member states to vote in favour and the supporting member states to represent at least 65% of the total EU population. To date, more than two decades after GM crops were first grown commercially in the USA, only two GM crops have been approved for cultivation in Europe: MON810, a variety of insect‐resistant (Bt) maize developed by Monsanto, as well as some derivatives produced by local breeders under licence from Monsanto, and the Amflora potato produced by BASF. Amflora was engineered to contain starch consisting almost entirely of amylopectin, resulting from the silencing of a granule‐bound starch synthase gene by RNA interference,^1^ and was intended for the industrial starch market. It spent 10 years in the EU's approval process and, although it was finally approved in 2010, BASF pulled out of European Biotech in 2012 and Amflora is now not available anywhere.

This leaves MON810 and its derivatives as the only GM crop varieties available to EU farmers. MON810 has been grown in Spain since 1998, and approval for its cultivation across the EU was granted in 2004. The area of GM maize cultivation in Spain in 2016 was 129 081 ha,^2^ with approximately 7000 ha in Portugal and small areas of cultivation in Slovakia and the Czech Republic. These areas are tiny compared with the tens of millions of hectares of GM crops grown in the Americas and Asia, although the area in Spain has been growing in recent years.

Efforts to develop new GM crop varieties for cultivation in the EU or elsewhere in Europe have now been abandoned by biotech companies. Instead, companies are focusing on obtaining permission for food and feed use without seeking approval for cultivation, so that their potential customers elsewhere in the world can be reassured that the European market is open to their products. This means that European farmers are competing with GM crops but are unable to grow them.

## DEFINITIONS

Defining what GM means in the context of crop biotechnology is not as easy as might be thought. In Directive 2001/18/EC, a genetically modified organism is defined as one in which the genetic material has been altered in a way that does not occur naturally. This raises problems because some of the techniques used in ‘traditional’ plant breeding and modern genomics produce genetic changes that would not occur naturally. Radiation and chemical mutagenesis, for example, produce thousands of mutations in an individual plant. The products of these treatments are considered GMOs as defined by the Directive, but are granted a ‘mutagenesis exemption’, mainly because varieties of crops with artificially induced mutations were already widespread and had been for decades when the Directive was drawn up. Oilseed rape, for example, was only made fit for human consumption through an intensive programme of mutagenesis in the second half of the 20th century that reduced the levels of erucic acid and glucosinolates, both of which are toxic. Mutation does occur naturally, of course (all of our major crops differed greatly from their wild ancestors long before the advent of modern plant breeding, in the main as a result of the selection of individuals carrying naturally occurring mutations), but not at anything like the rate induced by chemical or radiation mutagenesis.

More recently, modern genomics techniques in plant breeding have exploited doubled haploid populations for the identification of quantitative trait loci and genetic markers for marker assisted breeding. The production of these populations involves forced crosses between species that would not naturally interbreed (wheat and maize, for example), followed by chemically induced chromosome doubling in haploid embryos that have lost the ‘alien’ chromosomes – not very ‘natural’. Even triticale, now a familiar crop, is a hybrid of wheat and rye that was created in a lab: wheat and rye do not ‘naturally’ form viable hybrids. Nevertheless, triticale and varieties of other crops produced by these methods do not come under the EU's GM legislation and can be marketed in the EU without going through the safety checks and regulatory hurdles applied to GM crops.

One possible definition of a GM crop might be that it contains DNA from a sexually incompatible species. There are many examples of this in current, highly successful GM crop varieties on the market today. For example, the 5′‐enolpyruvylshikimate phosphate synthase (*EPSPS*) gene used by Monsanto to engineer glyphosate tolerance in crops originated from *Agrobacterium tumefaciens*,^3^ while the *Cry* genes used to impart insect resistance in ‘Bt’ crops come from another bacterium: *Bacillus thuringiensis*.^4^ Such genes are called transgenes, and the organisms that receive them are referred to as transgenic. The question of the ethics of transferring genes between different species has been raised famously by Prince Charles, who has been quoted as saying that ‘Mixing genetic material from species that cannot breed naturally, takes us into areas that should be left to God.’ Whatever we think about that statement, genetic modification does not necessarily involve the transfer of DNA between species. For example, potato varieties are now on the market in the USA carrying multiple traits imparted by inserted genes derived entirely from potato. The varieties have been developed by the JR Simplot Company of Boise, Idaho, and are marketed as Innate^®^ and Innate^®^ Generation 2. Both varieties have reduced expression in the tubers of an asparagine synthetase gene, *ASN1*, as well as reduced expression of two genes encoding enzymes of starch breakdown – phosphorylase L (*PhL*) and starch‐associated R1 (*R1*) – and a gene (*PPO5*) encoding polyphenol oxidase, an enzyme involved in bruising, all as a result of RNA interference. Innate^®^ Generation 2 also has reduced expression of vacuolar invertase (*VInv*) and increased resistance to late blight (*Phytophthora infestans*) through incorporation of the *Rpi‐vnt1.1* gene from *Solanum venturii*. The low concentration of free asparagine and reducing sugars in the tubers of Innate^®^ Generation 2 is claimed to reduce the formation of acrylamide by 90% compared with conventional potatoes. Acrylamide^5^ is a processing contaminant classed by the International Agency for Research on Cancer as a Group 2A carcinogen (probably carcinogenic to humans). Innate^®^ and Innate^®^ Generation 2 are described as cisgenic rather than transgenic, and this distinction has been prominent in the promotional material that has accompanied their release.

It has been argued that cisgenic plants should not come under GM regulations.^6^ If such a distinction between transgenic and cisgenic were to be made, the question would then arise as to how close a relative to a crop species must a source species be for a gene to qualify as a cisgene rather than a transgene. The late blight resistance gene, *Rpi‐vnt1.1*, that is present in Innate^®^ Generation 2, for example, originated from a wild potato: *Solanum venturii* 7 – definitely a potato species, but not the same species as cultivated potato: *Solanum tuberosum*. However, currently this is a moot point because the notion that cisgenic plants should not be regulated in the same way as transgenics has so far been rejected.^8^ Of course, whether cisgenic or transgenic, the introduced gene will have inserted into the host genome by DNA recombination, but DNA recombines naturally all of the time, so the presence of recombinant DNA itself cannot be used as a definition of a GMO.

Given all of the above, the European Commission cannot use its own official definition of a GMO (an organism in which the genetic material has been altered in a way that does not occur naturally) when considering whether a new crop variety should have to go through its approval process for GM crops, or define a GMO as an organism containing a foreign gene or recombinant DNA. It has therefore fallen back on defining a GMO based on the processes used to produce it. As far as GM plants go, that means plants regenerated from cells or protoplasts following *Agrobacterium tumefaciens*‐mediated transformation, plants grown from seeds that have been transformed by *Agrobacterium tumefaciens* using the floral dip method, plants arising from direct transfer of a gene into protoplasts using polyethylene glycol or electroporation, or direct transfer of a gene into plant material by particle bombardment or silicon carbide fibre vortexing. It was inevitable that this approach would cause problems as new technological advances were made.

## NEW METHODS, NEW PROBLEMS: GENOME EDITING

The last decade has seen the emergence of a raft of techniques that have been given the umbrella term of genome (or gene) editing because they are used to make mutations in specific target genes in a plant's genome. These technologies use specially designed oligonucleotides (oligonucleotide‐directed mutagenesis, or ODM), or targeted nucleases such as zinc‐finger nucleases (ZFN), meganucleases, transcription activator‐like effector nucleases (TALENs) or components of a bacterial defence system based on clustered, regularly interspaced, short palindromic repeats (CRISPR) and the Cas9 nuclease. Those involving targeted nucleases have up to now required a GM step to introduce a gene encoding the nuclease, and in the case of CRISPR‐Cas9 a guide RNA, into the host plant genome. However, once the mutation has been made the transgene can be eliminated by ‘selfing’ the edited plant and selecting progeny that carry the gene edit but not the transgene. Furthermore, techniques are being developed to introduce the nuclease directly into plant cells, bypassing the GM step. In their most common usage, genome editing techniques introduce a ‘knock‐out’ mutation in the target gene, in the form of a short deletion. More sophisticated applications are being developed, but certainly when used in its simplest form there is no scientific justification for treating a genome‐edited plant differently from a plant produced by other mutagenesis techniques, or a plant that has arisen through natural mutation.

That certainly appears to be the view of the US Department of Agriculture (USDA), which has stated that it will not regulate a mushroom that has been edited with CRISPR‐Cas9, the first case of a genome‐edited food crop that has come before it (albeit a fungus, not a plant). The USDA also issued a press release on 28 March 2018 stating that it does not have any plans to regulate plants produced by genome editing that are indistinguishable from those developed through traditional breeding methods, as long as they are not plant pests or developed using plant pests (https://www.usda.gov/media/press‐releases/2018/03/28/secretary‐perdue‐issues‐usda‐statement‐plant‐breeding‐innovation). Argentina has also developed policies for genome‐edited plants (Resolution No. 173/2015), essentially saying that new varieties will be assessed on a case‐by‐case basis, but that if there is no new combination of genetic material and no transgenes have been used the product will not be regulated as a GMO. In Canada, plants with ‘novel traits’ are covered by the Plant Protection Act (1990), regardless of the technology used to produce them, and the cultivation of a sulfonylurea‐tolerant oilseed rape variety produced by ODM was approved by the Canadian Food Inspection Agency as long ago as 2013 (http://www.inspection.gc.ca/plants/plants‐with‐novel‐traits/approved‐under‐review/decision‐documents/dd‐2013‐100/eng/1427383332253/1427383674669). In contrast, the European Commission has deferred a decision on the regulation of genome‐edited plants to an unspecified future date. This is extremely damaging because European plant breeders will not invest in the technology without clarity on the regulatory situation.

There are some grounds for optimism that the situation is about to change: in January 2018, an Advocate General of the European Court of Justice, which is responsible for interpreting EU law and ensuring its equal application across the EU, issued an opinion (Opinion Case C‐528/16) on proceedings brought by Confédération Paysanne, a French farmers' union, and others, who sought an annulment of the mutagenesis exemption. The Advocate General reasserted that organisms obtained by mutagenesis are GMOs within the meaning of Directive 2001/18/EC, but went on to state that the mutagenesis exemption applied to all organisms obtained by any technique of mutagenesis, on condition that they do not contain recombinant nucleic acid molecules. Ignoring the fact that all organisms naturally contain recombinant DNA, this judgement would appear to give the go‐ahead to the genome editing of crops, as long as no transgene is present in the edited plants. The matter will now be considered by a judge, or panel of judges, but the opinions reached by the court's Advocates General are usually very influential and are followed in the majority of cases.

## NEW TRAITS, NEW PROBLEMS: GM CROPS WITH DELIBERATELY ALTERED COMPOSITION

The European Commission also faces the prospect of having to adapt its processes to the risk assessment of GM crops, foods and feeds with deliberately altered composition. As described above, the Commission's current risk assessment strategy, as applied by EFSA's GMO Panel, is based on the principle of substantial equivalence, in which GM crops and their derived food and feeds are compared with a conventional, non‐GM comparator. That is fine when the GM trait carried by a variety imparts herbicide tolerance, or resistance to disease or insects, but recent years have seen the development of GM crop varieties that have received deliberate modifications to their composition. Mavera maize, for example, which was developed by Renessen, a joint venture between Cargill and Monsanto, combines a triple stack of input traits (resistance to corn rootworm and the European corn borer, and tolerance of glyphosate) with high lysine content as the cherry on top. The Vistive soybean variety developed by DuPont, the oil from which has increased levels of oleic acid and reduced polyunsaturated fatty acids, increasing its shelf life and making it more stable during high‐temperature processing,^9^ is another example, as are the Innate^®^ and Innate^®^ Generation 2 potato varieties discussed above.

The Amflora potato variety also came into this class of GM crops and, unlike the three examples above, was intended for cultivation in Europe. The difficulties that the European Commission had in risk assessing Amflora and the prospect of having to deal with an increasing number of crops with altered composition highlighted the need for robust, workable processes for the safety assessment of GM crops with altered composition.

Up to that point, EFSA's position was that ‘Where no comparator can be identified, a comparative risk assessment cannot be made and a comprehensive safety and nutritional assessment of the GM plant and derived food and feed itself should be carried out.’ A team of scientists (of which the author was a member) was commissioned to review this position and concluded that comparative approaches based on the concept of substantial equivalence were the basis of all current risk assessment strategies across the world, whether or not the composition of the crop had been deliberately altered.^10^, ^11^ Indeed, GM varieties with deliberately altered composition that had already been approved in the USA and other countries outside the EU had been risk assessed using the same framework as those with input traits, with no specific modifications to risk assessment criteria. It remains to be seen whether the European Commission will adopt this approach.

## DEVOLUTION OF DECISION‐MAKING TO MEMBER STATES

It has been extremely difficult to get agreement on GM crop approvals from all European Union member states ever since the process was brought under the control of the European Commission. The qualified majority voting system and the implacable anti‐GM position of some member states, which meant that they voted against any GM crop approval regardless of the recommendation of the EFSA GMO Committee, arguably made the process dysfunctional. Even when approvals were granted, individual member states continued to impose their own bans. Under EU law, member states were only allowed to impose bans for safety reasons, for example if a GM crop posed a risk to the environment in that member state that it did not pose elsewhere. As it was, member states were imposing bans without any evidence of safety issues, flouting EU treaties and putting them at loggerheads with the European Commission. In 2006, the World Trade Organization ruled that the EU's position was illegal and criticized the bans imposed by individual member states.^12^

In order to resolve this lamentable situation, the European Commission proposed that some of the decision making on GM crop and food issues should be devolved back down to national member state governments, and in March 2015 proposals were approved for a new system of GM crop authorizations. The new system allows member states to request, via the Commission, that a company applying for approval of a GM crop adjust the geographical scope of its application to exclude a particular member state or region. If the applicant refuses to adjust the geographical scope of its authorization, member states are allowed to proceed with national bans. In simple terms, this allows member states to opt out of allowing cultivation or import of a GM crop that has been approved at EU level, without giving a reason, effectively legalizing the previously illegal bans imposed by some member states. Almost immediately, 15 member states told the European Commission that they would send territorial exclusion requests for any application to market a GM crop for cultivation. These countries were Austria, Bulgaria, Croatia, Cyprus, Denmark, France, Germany, Greece, Hungary, Italy, Latvia, Lithuania, the Netherlands, Poland and Slovenia. In addition, Scotland, Northern Ireland and Wales said they would opt out on a regional basis, as did Wallonia (the French‐speaking region of southern Belgium).

## WHAT IS THE EU MISSING OUT ON?

The worldwide area of land planted with GM crops in 2016 was 185 million hectares,^2^ representing approximately 12% of total world agriculture. More than three‐quarters (78%) of global soybean production was GM, with figures of 64%, 26% and 24% for cotton, maize and oilseed rape, respectively. Other crops with some GM varieties being grown commercially included sugar beet, alfalfa, papaya and poplar. GM crops were grown in 26 countries, with the USA, Brazil, Argentina, Canada, India, Paraguay, Pakistan, China, South Africa, Uruguay and Bolivia all planting more than a million hectares of GM crops. In contrast, only 136 000 ha were planted in the whole of the EU, despite the fact that the EU imported tens of millions of tonnes of GM soybean, maize and other crops for use in animal feed.

The most popular traits introduced by GM are undoubtedly herbicide tolerance and insect resistance, but there are also traits imparting virus or fungal/oomycete disease resistance, and traits affecting fruit ripening, oil content, starch composition, resistance to bruising, nutritional value and food safety. The Innate^®^ Generation 2 potato variety discussed already in this article shows where crop biotechnology is heading in the USA, with multiple quality traits as well as resistance to late blight disease, and improved consumer safety as a major selling point. There is currently no prospect of similar varieties being grown in the EU, highlighting how far behind crop biotechnology is in Europe compared with the USA, and how this is beginning to compromise efforts to improve food safety.

## CONCLUSIONS

It is highly unlikely that any biotech company will attempt to develop a GM crop for cultivation in the EU while the current regulatory system is in operation. This is not only affecting the competitiveness of European agriculture but, because the EU is the biggest commodity market in the world, EU regulations and European attitudes are a wet blanket for countries that supply it, with many African countries that would benefit hugely from GM crops afraid to adopt them for fear of losing access to the EU market.

It is vital that European attitudes change and over‐regulation is rolled back, because agriculture faces huge challenges in the coming decades, including inadequate supplies of fresh water, the advent of peak oil (the point when the maximum rate of global petroleum extraction is reached, after which the rate of production enters terminal decline), competition for land use, soil erosion, salination and pollution. Plant breeding will not be able to play its full part in meeting these challenges if some technologies are rendered unusable for no rational reason. However, it is difficult to see where the drive for change will come from.
